# On the Relationship between Energy Metabolism, Proteostasis, Aging and Parkinson’s Disease: Possible Causative Role of Methylglyoxal and Alleviative Potential of Carnosine

**DOI:** 10.14336/AD.2016.1030

**Published:** 2017-05-02

**Authors:** Alan R. Hipkiss

**Affiliations:** Aston Research Centre for Healthy Ageing (ARCHA), School of Health and Life Sciences, Aston University, Birmingham B4 7ET, United Kingdom.

**Keywords:** Parkinson’s, methylglyoxal, glycolysis, mitochondria, proteolysis, glycation, homeostasis, carnosine, aging

## Abstract

Recent research shows that energy metabolism can strongly influence proteostasis and thereby affect onset of aging and related disease such as Parkinson’s disease (PD). Changes in glycolytic and proteolytic activities (influenced by diet and development) are suggested to synergistically create a self-reinforcing deleterious cycle via enhanced formation of triose phosphates (dihydroxyacetone-phosphate and glyceraldehyde-3-phosphate) and their decomposition product methylglyoxal (MG). It is proposed that triose phosphates and/or MG contribute to the development of PD and its attendant pathophysiological symptoms. MG can induce many of the macromolecular modifications (e.g. protein glycation) which characterise the aged-phenotype. MG can also react with dopamine to generate a salsolinol-like product, 1-acetyl-6,7-dihydroxy-1,2,3,4-tetrahydroisoquinaline (ADTIQ), which accumulates in the Parkinson’s disease (PD) brain and whose effects on mitochondria, analogous to MPTP (1-methyl-4-phenyl-1,2,3,6-tetrahydropyridine), closely resemble changes associated with PD. MG can directly damage the intracellular proteolytic apparatus and modify proteins into non-degradable (cross-linked) forms. It is suggested that increased endogenous MG formation may result from either, or both, enhanced glycolytic activity and decreased proteolytic activity and contribute to the macromolecular changes associated with PD. Carnosine, a naturally-occurring dipeptide, may ameliorate MG-induced effects due, in part, to its carbonyl-scavenging activity. The possibility that ingestion of highly glycated proteins could also contribute to age-related brain dysfunction is briefly discussed.

The objective of this opinion piece is to offer possible answers to the question “why is ageing a risk factor for Parkinson’s disease?” It is suggested that (i) excessive glycolysis accelerates aging onset caused in part by increased generation of the triose phosphates dihydroxyacetone-phosphate and glyceraldehyde-3-phosphate, and their decomposition product methylglyoxal (MG), (ii) MG causes mitochondrial dysfunction and provokes a compensatory, but eventually deleterious, increase in glycolysis, (iii) the developmentally-related decline in proteostasis (cytosolic and mitochondrial) synergistically induce a self-reinforcing deleterious cycle of dysfunction which increase the risk of aging generally and PD in particular, and (iv) increased MG accumulation in substantia nigra promotes formation of a neurotoxin found in the brains of PD patients and diabetics.

## Glycolysis is a risk factor

It is becoming increasingly apparent that the glycolytic pathway is not a benign sequence of reactions [1,2]. There is considerable evidence showing that aging, in many species, can be delayed by dietary interventions which impact particularly on glycolysis. Studies in various organisms show that lifespan can be increased when glycolytic flux is partially inhibited [3], due to either permanent caloric restriction [4] or every-other-day feeding (i.e. intermittent fasting) [5,6], deficiency in insulin-signalling [3,7], and treatment with either a non-metabolisable glucose analogue (2-deoxyglucose) [8] or the mTOR inhibitor rapamycin [9-11]. Although the mechanism(s) responsible for these effects is/are still debated, there is increasing evidence that decreased generation of methylglyoxal (MG), arising because of lowered glycolytic activity, could, at least in part, explain the beneficial effects on lifespan and onset of much age-related dysfunction and pathology [2,12-15].

MG is a by-product of glycolysis, formed predominantly by the spontaneous decomposition of the triose-phosphates glyceraldehyde-3-phosphate and dihydroxyacetone-phosphate, both of which are normal glycolytic intermediates; MG can also arise from catabolism of the amino acids serine and threonine and from lipid peroxidation. Animal studies have shown that tissue levels of MG are strongly raised following high glycemic-index meals [16,17], hyperglycaemic episodes [18] and intake of high levels of fructose [19]. Furthermore, MG levels in erythrocytes from diabetic patients have been shown to be increased at least 15-fold compared to controls, possibly due to decreased glyceraldehyde-3-phosphate dehydrogenase (GAPDH) activity [20,21]. It is also possible that catalytic function of the glycolytic enzyme triose-phosphate isomerase (TPI) may decline due to activity-induced asparagine deamidation; insufficient TPI activity could cause accumulation of dihydroxyacetone phosphate and contribute to increased MG generation following spontaneous decomposition of the triose phosphate [22,23]. Given that protein synthesis cannot occur in human erythrocytes, the possibility arises that excessive and persistent glycolysis due to constant hyperglycemia consequent upon almost continuous carbohydrate intake could lower TPI activity to such a degree that MG accumulates. This proposal therefore suggests that erythrocytes could be a major source of MG [24] during persistent hyperglycemia.

It is interesting to note that when the relative activities of the glycolytic enzymes are compared, TPI activity in erythrocytes is almost 4-fold greater than any other glycolytic enzyme [25]. Such elevated TPI activity may reflect an evolutionary adaptation to the likelihood of metabolically-induced decline in TPI activity during the erythrocyte lifespan, prior to its eventual removal after 120 days (in humans). However, as a result increased reliance upon carbohydrate, especially in the so-called Western diet, it can be argued that human diets have radically changed compared to food intake during the majority of human evolution. Consequently, it is possible that the initial excess of TPI within human erythrocytes is insufficient to compensate for the enhanced rate of TPI inactivation induced by the overall increase in glycolysis which occurs because of the Western diet. Such a proposal could contribute to the increased incidence of metabolic disease in which MG appears to play a predominant role.

It has been shown that fibroblasts obtained from PD patients possess defects in energy metabolism and proteostasis [26] and it is possible that increased MG generation contributes to both deficiencies. While an in-depth review of MG’s reactivity and biological effects is beyond the scope of this brief opinion piece, one can safely state that MG is a reactive bicarbonyl [27], whose action on cells and tissues mimics many of the effects of aging [24,28,29], and is therefore increasingly regarded as causal to much age-related dysfunction including neurodegeneration [30,31]. The effect of MG on proteins is the formation of advanced glycation end-products (AGEs) following its reaction with lysine, arginine, histidine and cysteine residues [32]; the resultant glycated proteins become resistant to intracellular proteolysis if they become cross-linked [33]. Not only can MG promote formation of undegradable proteins but the bicarbonyl can also inhibit intracellular proteolytic activity towards other aberrant proteins [34,35,36]. MG can directly compromise ubiquitin function by reacting with lysine residue 78, thereby inhibiting attachment of further ubiquitin molecules [17]. MG can also react with mitochondria to provoke organelle dysfunction accompanied by increased ROS generation [13,28,37,38]; one likely effect is a compensating increase in glycolysis in order to maintain sufficient ATP synthesis, but which exacerbates the situation by increasing MG formation.

## Methylglyoxal and PD

There is increasing evidence to suggest that excess carbohydrate catabolism may be causal to PD [39,40], possibly due to raised levels of MG. Dunn et al. proposed that an early event in spontaneous PD is dysregulation of glucose metabolism [41]. There are also reports that diets which are of low glycemic index may decrease PD risk [42-46]. Interestingly, the protein Parkin, a mutation in which increases PD risk, strongly affects energy metabolism: overexpression of Parkin inhibits glycolysis and stimulates mitochondrial activity, whilst loss of Parkin function results in decreased mitochondrial activity and increased glycolysis [47,48,49,50]. Additionally, it has been found that PINK1 deficiency (implicated in familial PD onset) stimulates glycolysis via stabilization of hypoxia-inducible factor-1alpha (HIF1-alpha) [51].

It is also interesting to note that PD is frequently associated with increased protein glycation [28,31,52,53] especially of α-synuclein which accumulates in PD brain as Lewy bodies [30,49,54,55]. Additionally, it has been shown that mutation in the DJ-1 gene is a PD risk factor: a study by Shi et al. has shown that DJ-1 deficiency activates glycolysis in muscle by inducing a Warburg-like metabolic reprogramming [56]; while another recent study showed that loss of DJ-1 function disrupts mitochondrial function, whereas DJ-1 activity represses glycolysis [57]. Interestingly a member of the DJ-1 superfamily (Hsp-31) has been reported to repair proteins modified by MG [58].

Glyoxalase-1 is an important enzyme for MG destruction [59,60] and glyoxalase-1 activity has been found to decline with age in rodent tissues [61], during senescence of cultured human fibroblasts [62] and in the human brain [63], while changes in glyoxalase-1 activity in *C. elegans* has been shown to strongly influence both mitochondrial function and organism lifespan [64].

Not only can MG directly damage mitochondria [65,66] and may be associated with age-related cognitive decline [67], but the bicarbonyl can also react with dopamine [68,69] to form 1-acetyl-6,7-dihydroxy-1,2,3,4-tetrahydro-isoquinaline (ADTIQ), which is a salsolinol-like compound [69]. ADTIQ not only accumulates in PD brains but also mimics the effects of MPTP (1-methyl-4-phenyl-1,2,3,6-tetrahydropyridine) whose effects on mitochondria resemble the changes associated with PD [69]. Further evidence supporting the proposed link between MG, dopamine and generation of the neurotoxin ADTIQ is shown by the increased levels of glycolytic enzymes, MG and ADTIQ in the brains of diabetic rats [70]. It may be relevant to note that another group of nigrostriatal neurotoxins, N-methylated-beta-carboline and 2,9-dimethyl beta-carboline, whose structures resembles MPTP, not only inhibit mitochondria respiration but are also potent inhibitors of triosephosphate isomerase (TPI) [71]. Inhibition of TPI provokes accumulation of dihydroxyacetone-phosphate which is not only a strong glycating agent but spontaneously decomposes into MG and has been proposed to contribute to neurodegeneration associated with Alzheimer’s disease [72,73]. As outlined above, it is also possible that TPI activity could decline due to over-use of the enzyme inducing deamidation of two asparagine residues in the enzyme (a wear and tear phenomenon) [22,23], ultimately resulting in dihydroxyacetone-phosphate accumulation and increased MG formation.

In a study using mice fed a high glycemic-index diet, it was found that protein damage was increased in the retina and in the substantia nigra (presumed to be induced by MG or other endogenous glycating agents) up to 34-fold compared to animals fed a low glycemic-index diet [17]; however, this remarkable observation has been neither confirmed nor refuted in other laboratories studying the effect of dietary glycemic-index on brain protein modification. Notwithstanding this caveat, it reasonable to at least consider whether metabolic changes which influence aging via a raised potential for MG accumulation will, by reacting with dopamine, not only decrease dopamine levels in the substantia nigra but also increase the likelihood of further mitochondrial dysfunction and enhance the generation of those changes which characterise PD.

Although MG is regarded as a potential source of protein dysfunction, principally via glycation, it is uncertain whether direct MG ingestion can result in its survival and transport across the gut wall in sufficient quantities to provoke dysfunction [74,75]. In contrast, however, other studies have shown that diets containing high levels of glycated proteins (produced by MG treatment) can not only suppress the lifespan-extending effects of dietary restriction in mice [76], but also deleteriously affect memory in older humans [77,78].

Interestingly, there are hints in the literature suggesting a link between PD and AGE receptors (RAGEs): RAGE ablation or deficiency is protective towards murine dopaminergic neurons in a MPTP model of PD [79,80], and certain RAGE polymorphisms are associated with either susceptibility or protection against PD in Chinese Han populations [52]. The flavenoid myricitrin, which has been claimed to exert anti-PD activity, also alleviates MG-induced mitochondrial dysfunction, AGE formation and RAGE expression [81]. These findings support the present contention that MG may play an important role in PD causation.

That dietary glycated proteins, but not, perhaps, free dietary MG [74,75], are reported to be deleterious, raises the controversial possibility that intact glycated proteins could bind to RAGEs present on cells of digestive tract, thereby traverse the gut wall, to enter the enteric nervous system and then transfer to the CNS [82,83]. Furthermore, it is known that certain misfolded proteins, such as α-synuclein, can undergo cell-to-cell tranmission, analogous to prion proteins [84,85,86]. Whilst such a scenario may be regarded as somewhat speculative, these observations raise important and possibly alarming questions regarding the presence of highly glycated proteins in human diets with respect to neurodegenerative conditions, especially given recent findings of the effects of dietary glycated proteins on memory in elderly humans [78].

## Aging, proteostatic dysfunction and PD

Changes in protein quality control (via the ubiquitin/proteasomal, autophagic and mitophagic systems, together with chaperone proteins etc.) contribute to both aging generally and PD in particular [87-90]. Vulnerability to proteostatic dysfunction may increase with age due to the following scenario. As growth ceases when adulthood is approached, the rate of protein biosynthesis declines. It is suggested that the synthesis of the many necessary proteases, chaperone proteins and other factors required to ensure maintenance of protein quality [91], is likely to decline co-ordinately with ribosome numbers and overall protein biosynthesis rates [92]. (This has been observed in the bacterium *Escherichia coli* - see below). The nascent polypeptide-associated complex (NAC) is a eukaryotic chaperone protein complex which recognises misfolded proteins while still associated with the ribosome; NAC normally exists in equimolar levels with ribosomes, binding in a 1:1 ratio [93]. Consequently, any decline in the synthesis of NAC and other necessary chaperones etc. will lead to a decrease in the maximum available activity for the recognition and proteolytic elimination of aberrant proteins of any origin i.e. those arising because of biosynthetic errors, as well as those damaged post-synthetically by ROS or glycating agents such as MG. That chaperone proteins, e.g. hsc70 and hsp27, can become increasingly glycated with age or under hyperglycemic conditions [94,95], as also can ubiquitin [17], are further complicating factors which will contribute to proteostatic decline [96]. Thus, the developmentally-induced relative deficit of proteostatic capacity may well explain the increased vulnerability of adult organisms to age-related dysfunction, including accumulation of altered protein species, as well as increased PD risk. This condition would be further exacerbated should the homeostatic proteases and chaperone proteins themselves be inactivated by endogenous toxic agents such as MG. It is interesting to note that the naked mole-rat, a rodent which can live for over 30 years, unlike most rodents whose maximum lifespan is between 3 and 5 years, does not show an age-related decline in intracellular proteolytic activity [97].

Changes in cytosolic proteolytic activity can also impact energy metabolism. The enzyme 6-phospho-2-kinase 2,6-bisphosphatase (Pfkfb3) generates fructose-2-6-bisphosphate; this enzyme is normally subject to continuous proteolytic degradation mediated by the ubiquitin/proteasome system, but if proteolysis of Pfkfb3 is inhibited, in neurones for example, glycolysis is stimulated and oxidative stress is increased, resulting in neurodegeneration [98,99]. Explanations of this phenomenon include decreased glutathione synthesis and increased MG formation due to the increased triose-phosphate generation [23,100]. Thus, the decline in the ubiquitin/proteasomal system, which characterises PD may also contribute to increased MG formation in the PD brain. Furthermore, in the substantia nigra increased MG generation would (as previously mentioned) following reaction with dopamine, increase the likelihood of formation of ADTIQ, which has been detected in diabetic brains, which in turn will damage mitochondria etc. and further exacerbate the condition, to promote a self-reinforcing deleterious cycle of dysfunction [70,101].

Changes in mitochondrial proteostasis could play a role in both PD and aging [87,88]. Again, developmental changes may impact on mitochondrial proteostatic function, especially should the rate or frequency of intra-mitochondrial protein synthesis decline as organism or organelle growth rate decreases. It has been clearly demonstrated that the Lon protease is partly responsible for the selective elimination of damaged proteins within mitochondria and whose expression is linked with mitogenesis. However, the activity of this protease has been shown to decline with age in a variety of model aging systems and the Lon protein is itself subject to oxidant-induced inactivation [102,103]. It has also been claimed that loss of mitochondrial proteolytic activity can have deleterious effects upon cytosolic proteasomal activity [104,105], thereby illustrating the interdependence of the components of the cellular proteostatic apparatus, although details of the mechanism responsible are not fully understood. Dysfunction or knock down of Hsc70-5/mortalin, which plays a role in mitogenesis and mitochondrial proteostasis, is lethal to dopamine-producing neurones in a Drosophila model of PD [106]. A possible relationship between mitochondrial ribosome numbers and proteostasis is shown by the protein humanin, a putative translation product of mitochondrial 16S ribosomal RNA, which appears to suppress apoptosis, delay diabetes and inhibit amyloid-beta peptide toxicity [see 107 and refs therein].

It may be relevant to note in the bacterium *Escherichia coli* (bacteria being evolutionary precursors to mitochondria) it was found that organism growth rates strongly influenced constitutive ability to degrade aberrant polypeptide species in an exponential manner: constitutive proteolytic activity towards abnormal proteins approximately squared as growth rates doubled [108], as do ribosome numbers. Should this relationship hold for eukaryotic cells in either or both the cytosolic and mitochondrial compartments, it follows that, as growth rates or mitogenesis decline as adult size is approached, maintenance of proteostasis will be strongly influenced by previous cellular history (i.e. protein synthesis rate) and the half-lives of the components of the proteostatic apparatus, thereby creating a delayed decline in proteolytic activity. Such a hysteresis-like phenomenon could help to explain (i) the extended latency of many age-related conditions, including PD, where altered proteins rarely accumulate during periods of rapid growth, but do so when growth has essentially ceased, and (ii) why increased aerobic activity which in turn induces mitogenesis is frequently beneficial by delaying age-related dysfunction.

## Endogenous control of MG-mediated damage

It is argued above that MG may play important causal roles in both aging and PD, and that increased MG formation may occur as a consequence of chronic excessive glycolysis resulting from intake of high glycemic-index diets and/or compromised mitochondria-mediated ATP synthesis inducing a compensatory increased glycolytic flux to maintain ATP levels. In the event of increased MG formation, cells normally possess a defence apparatus, the glyoxalase system, which eliminates the reactive bicarbonyl converting it to d-lactate [see 28 for authoritative review]. However, it has been shown that glyoxalase activity declines with age [20,109], while lowered glyoxalase activity promotes mitochondrial dysfunction, accelerates aging and decreases lifespan in C. elegans, whereas upregulation is protective towards mitochondria and increases lifespan [64]. Defects in the DJ-1 gene are known PD risk factors, and a member of the DJ-1 superfamily whose expression is controlled by DJ-1, has recently been shown to catalyse the deglycation of MG-modified proteins [58].

Another possible and naturally-occurring protective agent may be the dipeptide carnosine (beta-alanyl-L-histidine). Not only can carnosine protect proteins against MG-mediated modification and inhibit the cross-linking of MG-modified protein with a normal polypeptide in model systems [110,111], but it can delay senescence in cultured cells [112], and inhibit growth of tumour cells [113] most likely by interfering with glycolysis [114]. Other studies suggest that carnosine exerts anti-oxidant activity [115,116], chelates toxic metal ions [117,118] and is protective towards a number of reactive sugars and aldehydes such as glucose, fructose, ribose, malondialdehyde, acetaldehyde, formaldehyde, dihydroxyacetone [119-122] and 4-hydroxy-nonenal [123]. By scavenging MG carnosine could also conceivably suppress generation of the neurotoxin ADTIQ [124].

Carnosine is subject to degradation into its constituent amino acids by serum and cellular carnosinases, CNDP1 and CNDP2 respectively [125-128]. However, it is interesting to note that brain carnosinase activity increases with age [129] while in PD brains overexpression of substantia nigra CNDP2 has been reported, compared to normal controls [130].

Animal studies indicate that carnosine is beneficial towards senescence-accelerated mice [131] and may have therapeutic potential where deleterious aldehydes such as MG appear to be important in age-related pathologies [132] such as diabetes [127,133,134], diabetic kidney disease [134-136], atherosclerosis [137-139] and cataractogenesis [140-144] as well as models of stroke [145-148], Alzheimer’s disease [149-151] and PD [152-154].

A number of double-blind, placebo-controlled dietary, supplementation studies have shown that carnosine improves aspects of brain function in human subjects. For example, behaviour was improved in autistic spectrum children [155], cognition was improved in schizophrenic patients [156], exercise performance and quality of life were improved in chronic heart disease patients [157] and there was a significant improvement in cognition in Gulf war disease veterans [158]; elderly patients fed carnosine plus the related peptide ansererine also showed improved cognition [159]. These observations imply that the presence of serum carnosinase is not necessarily an impediment to carnosine’s potential efficacy, at least as far as the brain is concerned. Indeed, manipulation of cellular carnosine levels by either enhancing dipeptide synthesis or suppression of carnosinase activity could be fruitful areas of enquiry.

## Concluding remarks

Homeostatic dysfunction (e.g. in proteostasis and mitochondrial maintenance) contributes causally to aging and related pathologies such as PD. Indeed, it has been shown that fibroblasts obtained from PD patients possess proteolytic and bioenergetic deficits [26]. It is possible that mitochondrial dysfunction (of any origin) may result in increased glycolytic activity to compensate for the decline in ATP generation, which will in turn increase the potential for generation of the triose-phosphates glyceraldehyde-3-phosphate and dihydroxyacetone-phosphate and their decomposition product MG. Alternatively, excessive glycolytic activity, perhaps induced by high glycemic-index diets, may increase MG formation, especially in erythrocytes, which then, following secretion, promotes mitochondrial dysfunction in various tissues thereby creating a deleterious self-reinforcing cycle of dysfunction, especially if glyoxalase activity is insufficient to eliminate the increased MG formation. Consequently, the rates at which endogenous toxins, such as MG and its triose-phosphate precursors, as well as endogenous protective agents (e.g. glyoxalase and carnosine) are either generated or removed, need to be carefully regulated if organism viability is not to be compromised.

The degree of age-related vulnerability varies somewhat between tissues and individuals, most probably due to differences in development and metabolism, environmental effects, including diet and life-style choices, as well as genetic influences, otherwise we would all submit to the same age-related disease at the same time. And, as pointed out in the present piece, regulation of the common pathway for provision of energy and metabolic intermediates (i.e. glycolysis) and possibly the ingestion of glycated proteins, as well as any accompanying developmentally-related decrease in proteolytic activity, are important for maintenance of viability: homeostasis must be maintained to prevent accumulation of MG and other potential glycating agents (e.g. triose-phosphates) and their possible participation in the development of age-related disorders such as PD. (See Table 1 for concise summary).

**Table 1:** Summary of factors which may either provoke or ameliorate age-related changes which contribute to Parkinson’s disease onset

Possible common causal factors or processes to Parkinson’s disease and aging
Endogenous synthesis of methylglyoxal (MG): possible causes
Excessive glycolysis
High glycemic index diet
Inactivation of triose-phosphate isomerase
Decline of MG-scavenging or MG-eliminating processes
Effects of MG include
Mitochondrial dysfunction
Proteostastic dysfunction
Protein cross-linking
Protein AGEs
Formation of ADTIQ (neurotoxin)
Increased intake and possible effects of dietary protein-AGEs
Reaction with RAGEs in gut wall
Cell to cell transmission to CNS (??)
Induction of cognitive dysfunction (??)
Possible ameliorative strategies towards aging and PD
Decreased glycolysis
Increased mitochondrial function
Low glycemic index diet
Increased intake or synthesis of carnosine (an anti-glycating/MG-scavenging agent).
Increased intake of leafy plant tissues containing anti-glycating/MG scavenging agents.
Raised glyoxalase activity.

This idea, in a more general sense, was most certainly better put, more than a quarter of a century ago, by the eminent cell-biologist and gerontologist Leonard Hayflick [160], who made the following comments about aging and its causation. Hayflick wrote “Why do we age?” may be the wrong question. The right question may be “Why do we live as long as we do”? Hayflick also prefaced his piece by two quotations: “The constancy of the *milieu interieur* is the condition for a free life” - Claude Bernard” and “The inconstancy of the *milieu interieur* is the condition for the finitude of life - George Sacher”. Much recent research confirms Hayflick’s prescience. While there are many ways of “getting dead”, those processes which maintain homeostasis are likely to be important for delay of age-related pathology and aging generally.
